# Late-life food insecurity and cognition: exploring timing, duration, and mechanisms among older Mexican adults

**DOI:** 10.1186/s12877-023-04497-7

**Published:** 2023-11-30

**Authors:** Joseph Saenz, Jaqueline C Avila

**Affiliations:** 1 Edson College of Nursing and Health Innovation, Arizona State University, 500 N 3rd Street, Phoenix, AZ 85004 USA; 2 Department of Gerontology, University of Massachusetts Boston, Boston, MA 02125 USA

**Keywords:** Food Insecurity, Cognition, Aging, Mexico, Latin America, MHAS, Depression, Health disparities

## Abstract

**Background:**

Food insecurity (FI) remains a global public health problem. FI is more prevalent in low-and middle-income countries than high-income countries. FI is related with worse cognitive outcomes including cognitive function, cognitive decline, and cognitive impairment. Few studies have sought to identify how patterns of FI relate with cognitive function in old age and the potential mechanisms underlying this association.

**Methods:**

Data from the 2015 and 2018 waves of the Mexican Health and Aging Study (n = 9,654, age 50+) were used in this study. Reports of FI in 2015 and 2018 were combined to create four patterns of FI groups: “persistently food secure”, “became food secure”, “became food insecure”, and “persistently food insecure”. Linear regression was used to estimate associations between patterns of FI and cognitive task performance. The mediating roles of depressive symptoms, body mass index, and chronic conditions were tested using Karlson, Holm, and Breen methodology.

**Results:**

Approximately half of the sample were persistently food secure, 17% became food secure, 14% became FI, and 15% experienced persistent FI. When adjusting for demographic/socioeconomic confounders, persistent FI related with worse Verbal Learning, Verbal Recall, Visual Scanning, and Verbal Fluency performance compared to the persistently food secure. Becoming FI related with worse Verbal Learning, Visual Scanning, and Verbal Fluency. Mediation analyses provided support for depressive symptoms mediating associations between FI and poorer cognition, where 48% of the association between persistent FI and worse Verbal Recall performance was attributed to higher depressive symptoms. Becoming food secure was not associated with cognitive performance compared to the persistently food secure.

**Conclusions:**

FI may represent an important modifiable risk factor for poorer cognitive outcomes among older adults. Public health efforts should focus on providing stable food access to older adults, especially those living in poverty.

## Background

Mexico has experienced dramatic population aging over the last century. Life expectancy has increased alongside fertility declines leading to a growing proportion of the population being age 65+ [1, 2], a trend that will continue in coming decades with the proportion of people aged 65 + expected to triple by 2050 to around 1 in 5 [3]. This will be accompanied by a sharp increase in the Mexican population living with dementia, which is projected to reach nearly 3.2 million by 2050 [4]. Population aging has driven research interest in how socioeconomic inequality and poverty relate to health and cognition in older Mexican adults [5, 6]. Socioeconomic factors such as education, low early-life socioeconomic status, and limited income have been linked with poorer cognitive outcomes in Mexico [5, 7, 8]. Although fewer studies have evaluated effects of food insecurity, which is more commonly experienced by those living in poverty [3], research on health effects of food insecurity has been expanding recently in Mexico.

Food security was defined by the Food and Agriculture Organization (FAO) in 2009 as the status “when all people, at all times, have physical, social, and economic access to sufficient, safe, and nutritious food that meet their dietary needs and food preferences for an active and healthy life” [9]. Food insecurity is present when any of these concepts are not met. Cross-national comparisons of food insecurity show a significant increase in moderate or severe food insecurity around the world, from 22.6% in 2014 to 30.4% in 2020 [9]. The prevalence of food insecurity in Latin America in 2020 was 38.7%, placing the prevalence significantly higher than Europe (9.3%) and Northern America (7.8%) but lower than regions most affected by food insecurity such as Sub-Saharan Africa (66.2%) and Southern Asia (43.8%) [9]. Drivers of food insecurity around the world include food supply disruptions, climate variation and climate extremes that affect food systems, economic recessions, lower food purchasing power, and increasing cost of healthy diets [10]. Latin America especially saw increases in the cost of healthy diets between 2017 and 2019, which also corresponded to one of the highest increases in food insecurity in the region [10]. Other individual-level determinants of food insecurity in Latin America include being older, having children in the household, being widowed or separated, rural dwelling, low education, low household income, and being employed part-time or unemployed [11].

### Food insecurity, health, and cognition

Food insecurity is associated with worse health outcomes among older adults, including worse self-reported health and greater prevalence of diabetes, hypertension, and heart disease [12]. Older adults with food insecurity are also more likely to have depression, stress [13, 14], higher allostatic load [15], and functional or mobility limitations [16, 17]. Simultaneously, individuals with poorer health and disability are also more likely to experience food insecurity, independently of other risk factors [18–20]. Recent work has suggested that food insecurity may also negatively impact the brain and cognitive function [21, 22]. Studies have reported effects of food insecurity on outcomes spanning lower cognitive function [23–29], faster cognitive decline [30–32], mild cognitive impairment [33–37], and dementia [38, 39].

Although these studies suggest negative effects of food insecurity on cognition, several gaps remain unfilled. First, mechanisms underlying food insecurity-cognition associations have received little empirical testing. Food insecurity has been argued to impact cognition through its effect on depression as experiencing food insecurity relates with higher odds of depression and elevated depressive symptoms [13, 21, 40, 41] and depression is related with worse cognitive outcomes [42]. There is also evidence that depressive symptoms partially mediate relationships between food insecurity and poorer cognitive function [33, 43].

Food insecurity may also impact cognition through its effects on chronic disease and body mass index (BMI). Food insecurity correlates with higher risk of chronic diseases including diabetes [44, 45] and hypertension [44, 46], both of which are associated with worse cognitive outcomes [47, 48]. Abnormal BMI is also more frequent among individuals experiencing food insecurity. Food insecurity has been related with obesity for reasons such as overconsumption when resources are available and consumption of cheap and calorie rich foods among individuals experiencing food insecurity [49, 50]. However, severe food insecurity has also been related with risk of being underweight [39, 51, 52]. BMI abnormality (both underweight and obesity) are related with poorer cognitive outcomes [53].

Second, studies of food insecurity and cognition have often not been positioned to assess the episodic nature of food insecurity as households may cycle into and out of food insecurity [54]. Recent studies have made use of food insecurity reports over time to capture changes in food insecurity status to better capture timing and duration of food insecurity. Compared to being persistently food secure in old age, former food insecurity, incident food insecurity, and ongoing food insecurity have been related with higher odds of major depression [41] when evaluating two year patterns of food insecurity. Regarding cognition, individuals who became food insecure over five years or experienced ongoing food insecurity had lower cognitive function compared to their counterparts who experienced persistent food security over five years [43].

Patterns in food insecurity status are important to consider. Former food insecurity may have lasting effects on cognitive function even after individuals regain food security. For example, depression may connect food insecurity to diminished cognitive ability, but prior work finds that correlations between depression and cognition may persist over time. For example, individuals who have experienced depression in the past may continue to exhibit lower cognitive function even after recovering from depression [55–57]. Former food insecurity may have lasting effects on cognition as food insecurity can lead to an increased risk of depression that persists over time [41]. Evaluation of patterns of food insecurity also allows for testing whether duration of food insecurity matters. Those who are currently experiencing food insecurity may be broken down into those who are experiencing new food insecurity and those for whom food insecurity is an ongoing problem. Differences between these groups may be expected given that prolonged exposure to food insecurity can lead to increasing cumulative experiences of stress and cumulative stress exposure negatively impacts cognitive function [58].

Last, there has been limited research on food insecurity and cognition in Mexico or Latin America [7, 29]. The Latin American region of the world has higher levels of food insecurity when compared to North America, Eastern Europe, and Central Asia [59]. Using data from the Encuesta Nacional de Salud y Nutrición, 44.8% of Mexican households were food secure in 2018 with 31.0%, 14.9%, and 9.3% of households having mild, moderate, and severe food insecurity, respectively. The proportion of households having food insecurity increased during the COVID-19 pandemic [60]. Previous evidence among older Mexican adults has shown that food insecurity in early life (before age 10) was associated with lower Verbal Learning scores in later life, and food insecurity in older ages was also associated with lower Visual Scanning scores [29]. Further, food availability was a significant mediator in the relationship between income and cognition [7].

### Current study

The primary aim of this study is to assess the episodic nature of food insecurity by investigating how three-year patterns of late-life food insecurity status relate with cognitive function among older Mexican adults. The second aim of this study is to test the mechanisms through which food insecurity relates with cognitive function by testing whether depressive symptoms, chronic disease, and BMI mediate associations between patterns of food insecurity and cognitive function.

## Methodology

### Study participants

We use data from the 2015 and 2018 waves of the Mexican Health and Aging Study (MHAS) [61]. The MHAS is a large, longitudinal, household-based, nationally representative study of adults age 50 + and their spouses, regardless of age. The study began in 2001 with a sample of 15,186. Follow-up interviews have been conducted in 2003, 2012, 2015, and 2018. The MHAS maintained representation of the population age 50 + by adding respondents born 1952–1962 to the 2012 wave and born 1963–1968 to the 2018 wave. The MHAS is a sister study to the United States Health and Retirement Study and has been described in greater detail elsewhere [62]. We focus analyses on the 2015 and 2018 waves as the two most recently available waves at the time of analysis.

Beginning with 11,952 study participants interviewed in 2015 and 2018, we first excluded respondents who were not age 50 + in 2018 (n = 235) as our focus is on cognitive function among older adults. Second, we excluded participants who required a proxy in 2018 because cognitive performance evaluations were not applied to this group (n = 1,051). Third, we excluded 124 participants who did not have observed data on any dependent variable (cognitive tasks described below) and an additional 888 who did not have complete data on independent variables (described below). The final analytic sample consisted of 9,654 individuals.

### Cognitive function

We analyzed four cognitive tasks in 2018. Verbal Learning was assessed as the immediate recall of an eight-word list across three trials (the number of words correctly recalled across the three trials are summed to create a measure with a range of 0–24). Verbal Recall involved participants recalling the eight-word list after a delay (range: 0–8). For Visual Scanning, participants were given a sheet of paper containing multiple symbols and were given one minute to mark each occurrence of a target stimulus (range: 0–60). Verbal Fluency was ascertained through a one-minute animal naming task (range: 0–60). Higher scores represent better cognitive ability for all tasks. Further information on the MHAS cognitive measures is available in other work [63, 64]. Although other cognitive tasks have been assessed in the MHAS, we focus on these four as these measure cognitive domains that have been previously assessed in relation to food insecurity such as memory and executive function [28, 29, 31]. These tasks also do not require mathematical or literacy abilities, which is essential in evaluations of cognition in settings with lower formal education levels. These tasks also have adequate ranges and approximately normal distributions.

### Late-life food insecurity

Our classification of patterns of food insecurity was based on prior studies assessing health impacts of food insecurity [41, 43]. In each wave, food insecurity was determined at the household level through two questions. First, participants were asked: “In the last two years, have you always had enough money to buy the food that you need?” with a yes/no answer choice. Second, respondents who answered “no” and those who did not know or refused to answer the first question were asked whether, “At any time in the last two years, did you not eat or ate less than you wanted because there was not enough food in your home.” Reporting either not having enough money to buy food or not eating or eating less than one wanted during the past two years was considered food insecurity. We then used food insecurity status in 2015 and 2018 to identify four patterns of food insecurity: “persistently food secure” (not food insecure in 2015 and 2018), “became food secure” (food insecure in 2015 but not 2018), “became food insecure” (not food insecure in 2015, but food insecure in 2018), and “persistently food insecure” (food insecure in 2015 and 2018).

### Confounding variables

Prior studies have identified demographic factors, socioeconomic position, living arrangements, and social capital as potential determinants of food insecurity [59, 65]. We control for demographic factors including age, gender, marital status (married/partnered, widowed, or other), whether participants speak an indigenous dialect, and locality size as a measure of rural/urban dwelling, which was categorized as 100,000+; 15,000–99,999; 2,500 − 14,999; or < 2,500 residents. In addition to marital status, a count of residents in the household was used to capture living arrangements. Social capital was assessed as whether respondents can rely on friends and neighbors for their daily needs and activities (yes/no). Socioeconomic controls included years of education, income decile, and household wealth decile. Income included labor income, pension income, and income from other sources including from public institutions. Health status in 2015 was included as a confounding variable and captured using the number of chronic conditions endorsed by respondents (hypertension, diabetes, stroke, heart attack, cancer, and respiratory conditions). All confounding variables were obtained from the 2015 wave.

### Mediating variables

We included several potential mediators. Depressive symptoms were captured using a nine-item Center for Epidemiologic Studies – Depression scale [66] where respondents reported whether they experienced various symptoms of depression in the past week. The number of endorsed depressive symptoms was treated as a continuous variable. BMI was calculated using self-reported height and weight and categorized into underweight (BMI: <18.5), normal (reference, BMI: 18.5–24.9), overweight (BMI: 25-29.9), obese (BMI: 30+), or missing. Self-reported height and weight has been validated in the MHAS [67]. Last, the count of chronic conditions reported in 2018 was tested as an additional mediating variable. All mediating variables were obtained from the 2018 wave of data.

### Statistical approach

We first conducted descriptive analyses comparing cognitive, demographic, socioeconomic, health, and social characteristics by food insecurity patterns using ANOVA and chi-square tests. Second, we used linear regression to estimate associations between food insecurity patterns and cognitive function with standard errors clustered at the household level. For each cognitive task, a first model included the main effects of food insecurity adjusted for confounders. A second model added potential mediators. These models test whether food insecurity patterns relate with cognitive task performance, how mediating variables relate with cognitive task performance, and whether associations between food insecurity patterns and cognitive performance persist after controlling for mediating variables. For our main analyses we use the persistently food secure as the reference group as all other groups experience food insecurity at some point. However, we conducted additional analyses in which the reference group was changed to becoming food insecure, becoming food secure, and persistent food insecurity to test additional questions (e.g., whether persistent food insecurity is related with worse cognition compared to becoming food insecure). Normality of residuals was verified using kernel density plots and standardized normal probability (P-P) plots. We leave cognitive variables in their raw form for descriptive analyses, but convert cognitive outcome variables to Z-scores by standardizing the raw scores to a mean of zero and a variance of one to facilitate comparisons of parameters across regression models.

Third, we used Karlson, Holm, and Breen (KHB) methods to formally test for mediation [68]. The KHB approach is based on linear regressions but decomposes effects of food insecurity patterns on cognitive function into direct (unexplained by mediators) and indirect (explained by mediators) components while adjusting for control variables. The KHB method was chosen to test mediation as it is intuitive and allows for testing significance of indirect (mediation) effects, estimation of the percent of the total association between food insecurity patterns and cognition that is indirect, and allows analysis of categorical independent variables. All analyses were conducted using Stata 17 MP4.

## Results

### Descriptive results

Descriptive characteristics of the analytic sample by food insecurity group are provided in Table 1. Slightly over half of the sample were persistently food secure, having not reported food insecurity in 2015 or 2018. Some changed food insecurity status between waves, with 14% becoming food insecure and 17% becoming food secure. Persistent food insecurity was experienced by 15% of the sample. Significant differences in cognitive function were observed across food insecurity patterns for all cognitive tasks (*p* < 0.001 for all cognitive tasks). The persistently food secure had the highest mean scores across cognitive tasks and the persistently food insecure generally had the lowest mean scores, although differences between the persistently food insecure and becoming food insecure were small. The food insecurity pattern groups did not differ in terms of age or gender but did differ in terms of socioeconomic status. Individuals in households experiencing any type of food insecurity tended to have less education, income, and wealth. For instance, the persistently food secure had an average of 7.0 years of education compared to only 3.9 years of education in the persistently food insecure group. Food insecurity also appeared to be more prevalent in rural areas. Of the persistently food secure, only 16% lived in communities with fewer than 2,500 residents compared to 23%, 27%, and 29% of the became food secure, became food insecure, and persistently food insecure groups, respectively. The persistently food insecure group endorsed depressive symptoms most frequently (mean: 4.3) whereas the persistently food secure group reported the fewest depressive symptoms (mean 2.9). Relative to the persistently food secure, groups experiencing any period of food insecurity reported more chronic conditions.

**Table 1:** Cognitive, demographic, socioeconomic, and health characteristics of older Mexican adults (age 50+) by pattern of food insecurity (n = 9,654)

	Pattern of Food Insecurity								
	Persistently Food Secure (n = 5,225)		Became Food Secure (n = 1,649)		Became Food Insecure (n = 1,380)		Persistently Food Insecure (n = 1,400)		
	Mean	SD	Mean	SD	Mean	SD	Mean	SD	*p*
Cognitive Function¹									
Verbal Learning (Range: 0–24)	14.7	3.8	14.0	3.6	13.8	3.9	13.5	3.8	***
Verbal Recall (Range: 0–8)	4.4	2.0	4.1	2.0	4.1	2.0	3.9	2.1	***
Visual Scanning (Range: 0–60)	31.1	15.9	27.4	15.3	25.8	14.9	24.0	14.4	***
Verbal Fluency (Range: 0–60)	15.8	5.3	14.9	4.9	14.3	4.8	14.0	4.8	***
Confounding Variables									
Age	65.6	9.2	65.1	9.2	65.3	9.1	65.1	8.9	
Female (n, %)	3,064	58.6	1,011	61.3	843	61.1	855	61.1	
Years of Education	7.0	5.0	4.9	3.9	4.6	4.0	3.9	3.4	***
Locality Size: 100,000+ (n, %)	3,398	65.0	877	53.2	646	46.8	618	44.1	***
Locality Size: 15,000–99,999 (n, %)	600	11.5	208	12.6	220	15.9	199	14.2	
Locality Size: 2,500 − 14,999 (n, %)	418	8.0	189	11.5	140	10.1	184	13.1	
Locality Size: <2,500 (n, %)	809	15.5	375	22.7	374	27.1	399	28.5	
Married/Partnered (n, %)	3,597	68.8	1,118	67.8	965	69.9	948	67.7	
Widowed (n, %)	902	17.3	293	17.8	248	18.0	238	17.0	
Divorced/Separated/Never Married (n, %)	726	13.9	238	14.4	167	12.1	214	15.3	
Can Rely on Friends/Neighbors (n, %)	3,273	62.6	957	58.0	787	57.0	769	54.9	***
Household Residents	2.7	2.0	3.1	2.2	3.0	2.1	3.3	2.5	***
Speaks Indigenous Dialect (n, %)	299	5.7	156	9.5	100	7.2	145	10.4	***
Income Decile	5.0	3.1	3.9	2.7	4.0	2.8	3.4	2.5	***
Wealth Decile	5.0	2.8	3.9	2.7	4.3	2.8	3.7	2.8	***
Chronic Conditions (2015)	1.1	0.9	1.1	1.0	1.1	1.0	1.2	1.0	***
Potential Mediators									
Depressive Symptoms	2.9	2.4	3.5	2.6	3.9	2.6	4.3	2.8	***
Chronic Conditions (2018)	1.2	1.0	1.3	1.0	1.3	1.0	1.3	1.1	***
BMI: Normal (n, %)	1,420	27.2	414	25.1	392	28.4	370	26.4	***
BMI: Underweight (n, %)	49	0.9	19	1.2	24	1.7	15	1.1	
BMI: Overweight (n, %)	2,038	39.0	554	33.6	444	32.2	448	32.0	
BMI: Obese (n, %)	1,269	24.3	418	25.3	327	23.7	302	21.6	
BMI: Missing (n, %)	449	8.6	244	14.8	193	14.0	265	18.9	

Note: Authors own calculation using data from the Mexican Health and Aging Study. Food insecurity patterns are based on food insecurity status in 2015 and 2018 waves. All confounding variables come from the 2015 wave whereas mediators come from the 2018 wave. SD: standard deviation. ***p***-value testing differences in variables across food insecurity groups. ***p***-values are obtained from ANOVA tests for continuous variables and chi-square tests for binary and categorical variables. * indicates ***p*** < 0.05, ** indicates ***p*** < 0.01, *** indicates ***p*** < 0.001. BMI: body mass index. ¹ Higher scores represent better cognitive ability for all tasks. Verbal Learning scores represent the number of words immediately recalled from an eight-word list across three trials. Verbal Recall represents the number of words recalled from the eight-word list after a delay. Visual Scanning represents the number of correctly identified target stimuli. Verbal Fluency represents the number of animals named in one minute

### Regression results

Regression results by cognitive task are shown in Table 2. For Verbal Learning, in Model 1, which included food insecurity patterns and confounders, becoming food insecure (β: -0.05, 95% Confidence Interval [CI]: -0.11, -0.00) and persistent food insecurity (β: -0.07, 95% CI: -0.13, -0.02) were related with lower Verbal Learning performance relative to the persistently food secure but these associations were no longer statistically significant when adding mediators in Model 2. Regarding Verbal Recall, persistent food insecurity was related with worse scores in Model 3 before adjustment for mediators (β: -0.06, 95% CI: -0.12, -0.00), but this association was no longer statistically significant when adding mediators in Model 4.

**Table 2:** Results from regression models of cognitive task performance on food insecurity, confounders, and mediators among older Mexican adults

	**Verbal Learning**				**Verbal Recall**				**Visual Scanning**				**Verbal Fluency**			
	Model 1		Model 2		Model 3		Model 4		Model 5		Model 6		Model 7		Model 8	
	β	SE	β	SE	β	SE	β	SE	β	SE	β	SE	β	SE	β	SE
Food Insecurity (ref: Persistently Food Secure)																
Became Food Secure	-0.03	(0.03)	-0.02	(0.02)	-0.02	(0.03)	-0.00	(0.03)	-0.01	(0.02)	-0.00	(0.02)	-0.00	(0.03)	0.01	(0.03)
Became Food Insecure	-0.05*	(0.03)	-0.02	(0.03)	-0.01	(0.03)	0.01	(0.03)	-0.09***	(0.03)	-0.07**	(0.03)	-0.08**	(0.03)	-0.06*	(0.03)
Persistently Food Insecure	-0.07**	(0.03)	-0.03	(0.03)	-0.06*	(0.03)	-0.02	(0.03)	-0.11***	(0.03)	-0.08**	(0.03)	-0.07**	(0.03)	-0.04	(0.03)
Confounders																
Age	-0.03***	(0.00)	-0.03***	(0.00)	-0.03***	(0.00)	-0.03***	(0.00)	-0.04***	(0.00)	-0.03***	(0.00)	-0.02***	(0.00)	-0.02***	(0.00)
Female	0.30***	(0.02)	0.33***	(0.02)	0.31***	(0.02)	0.34***	(0.02)	0.01	(0.02)	0.03	(0.02)	-0.05*	(0.02)	-0.02	(0.02)
Years of Education	0.06***	(0.00)	0.06***	(0.00)	0.04***	(0.00)	0.04***	(0.00)	0.08***	(0.00)	0.08***	(0.00)	0.06***	(0.00)	0.06***	(0.00)
Locality Size 15k-99.9k vs. 100k+	-0.04	(0.03)	-0.04	(0.03)	0.01	(0.03)	0.01	(0.03)	-0.04	(0.03)	-0.04	(0.03)	-0.04	(0.03)	-0.04	(0.03)
Locality Size 2.5k-14.9k vs. 100k+	-0.13***	(0.03)	-0.11***	(0.03)	-0.06	(0.03)	-0.05	(0.03)	-0.19***	(0.03)	-0.18***	(0.03)	-0.08*	(0.03)	-0.07*	(0.03)
Locality Size < 2.5k vs. 100k+	-0.23***	(0.03)	-0.20***	(0.03)	-0.18***	(0.03)	-0.15***	(0.03)	-0.26***	(0.02)	-0.23***	(0.02)	-0.14***	(0.03)	-0.11***	(0.03)
Widowed vs. Married/Partnered	-0.05*	(0.03)	-0.04	(0.03)	0.00	(0.03)	0.02	(0.03)	-0.06*	(0.02)	-0.05	(0.02)	-0.06*	(0.03)	-0.05	(0.03)
Div/Sep/Nev vs. Married/Partnered	-0.02	(0.03)	-0.01	(0.03)	-0.02	(0.03)	-0.01	(0.03)	-0.03	(0.03)	-0.02	(0.03)	-0.01	(0.03)	0.00	(0.03)
Can Rely on Others	0.08***	(0.02)	0.07***	(0.02)	0.07***	(0.02)	0.06**	(0.02)	0.03	(0.02)	0.02	(0.02)	0.06**	(0.02)	0.05**	(0.02)
Household Residents	-0.00	(0.00)	-0.00	(0.00)	0.00	(0.00)	0.00	(0.00)	-0.01	(0.00)	-0.00	(0.00)	-0.00	(0.00)	-0.00	(0.00)
Speaks Indigenous Dialect	-0.27***	(0.04)	-0.24***	(0.04)	-0.07	(0.04)	-0.05	(0.04)	-0.24***	(0.03)	-0.21***	(0.03)	-0.33***	(0.03)	-0.31***	(0.03)
Income Decile	0.01***	(0.00)	0.01**	(0.00)	0.01***	(0.00)	0.01**	(0.00)	0.02***	(0.00)	0.01***	(0.00)	0.01**	(0.00)	0.01**	(0.00)
Wealth Decile	0.00	(0.00)	0.00	(0.00)	-0.00	(0.00)	-0.00	(0.00)	0.02***	(0.00)	0.02***	(0.00)	0.01***	(0.00)	0.01***	(0.00)
Chronic Condition Count (2015)	-0.02*	(0.01)	0.02	(0.02)	-0.03**	(0.01)	-0.00	(0.02)	-0.05***	(0.01)	-0.01	(0.02)	-0.03**	(0.01)	0.05*	(0.02)
Mediators																
Depressive Symptoms			-0.03***	(0.00)			-0.02***	(0.00)			-0.01***	(0.00)			-0.02***	(0.00)
Chronic Condition Count (2018)			-0.04*	(0.02)			-0.02	(0.02)			-0.04*	(0.02)			-0.07***	(0.02)
BMI: Underweight vs. Normal			-0.20*	(0.10)			0.01	(0.08)			-0.06	(0.07)			-0.20*	(0.10)
BMI: Overweight vs. Normal			0.11***	(0.02)			0.07**	(0.02)			0.09***	(0.02)			0.07**	(0.02)
BMI: Obese vs. Normal			0.12***	(0.02)			0.08**	(0.03)			0.09***	(0.02)			0.06*	(0.03)
BMI: Missing vs. Normal			-0.12***	(0.03)			-0.18***	(0.04)			-0.17***	(0.03)			-0.15***	(0.03)
Observations	9623		9623		9623		9623		8799		8799		9463		9463	

Note: Authors’ own calculation using data from the 2015 and 2018 Mexican Health and Aging Study. Verbal Learning, Verbal Recall, Visual Scanning, and Verbal Fluency are standardized. β indicates regression coefficient. SE indicates standard error. BMI: Body Mass Index. * indicates ***p*** < 0.05, ** indicates ***p*** < 0.01, *** indicates ***p*** < 0.001

Differences in cognition across the food insecurity patterns were observed for Visual Scanning (Models 5 and 6) with both becoming food insecure (β: -0.09, 95% CI: -0.14, -0.04) and persistent food insecurity (β: -0.11, 95% CI: -0.16, -0.06) related with worse Visual Scanning compared to persistent food security in Model 5. Becoming food secure was not related with Visual Scanning performance. When mediators were added in Model 6, becoming food insecure (β: -0.07, 95% CI: -0.12, -0.02) and persistent food insecurity (β: -0.08, 95% CI: -0.13, -0.03) remained statistically significant, but parameter estimates were reduced.

Verbal Fluency models also reflected a pattern of both becoming food insecure (β: -0.08, 95% CI: -0.13, -0.03) and persistent food insecurity (β: -0.07, 95% CI: -0.13, -0.02) being related with worse cognitive function relative to the persistently food secure when only adjusting for confounders (Model 7). When mediating variables were added in Model 8, becoming food insecure remained statistically significant but the parameter estimate was smaller (β: -0.06, 95% CI: -0.11, -0.00) and the estimate for persistent food insecurity was no longer statistically significant. Becoming food secure was not significantly associated with Verbal Fluency.

### Alternate reference groups

We then re-estimated models in Table 2 using different reference groups for food insecurity patterns and present these results in Table 3. Specification 1 uses “became food secure” as the reference. Specification 2 uses “became food insecure” as the reference. Specification 3 uses “persistently food insecure” as the reference. Compared to those who became food insecure (Specification 2), we find that persistent food insecurity was not associated with significantly worse cognitive function. This suggests that, among those currently reporting food insecurity, cognition was not significantly worse if the food insecurity was persistent. The importance of current reports of food insecurity relative to past food insecurity is also clear in Specification 1 where, compared to becoming food secure, both becoming food insecure and persistent food insecurity were related with worse Visual Scanning and Verbal Fluency performance.

**Table 3:** Results from regression models of cognitive task performance on food insecurity, confounders, and mediators among older Mexican adults: specification using alternate reference groups

	**Verbal Learning**				**Verbal Recall**				**Visual Scanning**				**Verbal Fluency**			
	Model 1		Model 2		Model 3		Model 4		Model 5		Model 6		Model 7		Model 8	
	β	SE	β	SE	β	SE	β	SE	β	SE	β	SE	β	SE	β	SE
Food Insecurity Specification 1 (Ref: Became Food Secure)																
Persistently Food Secure	0.03	(0.03)	0.02	(0.02)	0.02	(0.03)	0.00	(0.03)	0.01	(0.02)	0.00	(0.02)	0.00	(0.03)	-0.01	(0.03)
Became Food Insecure	-0.02	(0.03)	-0.01	(0.03)	0.01	(0.03)	0.02	(0.03)	-0.08*	(0.03)	-0.07*	(0.03)	-0.08*	(0.03)	-0.07*	(0.03)
Persistent Food Insecurity	-0.04	(0.03)	-0.02	(0.03)	-0.04	(0.03)	-0.01	(0.03)	-0.10**	(0.03)	-0.08**	(0.03)	-0.07*	(0.03)	-0.05	(0.03)
Food Insecurity Specification 2 (Ref: Became Food Insecure)																
Persistently Food Secure	0.05*	(0.03)	0.02	(0.03)	0.01	(0.03)	-0.01	(0.03)	0.09***	(0.03)	0.07**	(0.03)	0.08**	(0.03)	0.06*	(0.03)
Became Food Secure	0.02	(0.03)	0.01	(0.03)	-0.01	(0.03)	-0.02	(0.03)	0.08*	(0.03)	0.07*	(0.03)	0.08*	(0.03)	0.07*	(0.03)
Persistent Food Insecurity	-0.02	(0.03)	-0.01	(0.03)	-0.05	(0.04)	-0.03	(0.04)	-0.02	(0.03)	-0.01	(0.03)	0.01	(0.03)	0.02	(0.03)
Food Insecurity Specification 3 (Ref: Persistent Food Insecurity)																
Persistently Food Secure	0.07**	(0.03)	0.03	(0.03)	0.06*	(0.03)	0.02	(0.03)	0.11***	(0.03)	0.08**	(0.03)	0.07**	(0.03)	0.04	(0.03)
Became Food Secure	0.04	(0.03)	0.02	(0.03)	0.04	(0.03)	0.01	(0.03)	0.10**	(0.03)	0.08**	(0.03)	0.07*	(0.03)	0.05	(0.03)
Became Food Insecure	0.02	(0.03)	0.01	(0.03)	0.05	(0.04)	0.03	(0.04)	0.02	(0.03)	0.01	(0.03)	-0.01	(0.03)	-0.02	(0.03)

Note: Authors’ own calculation using data from the 2015 and 2018 Mexican Health and Aging Study. Verbal Learning, Verbal Recall, Visual Scanning, and Verbal Fluency are standardized. β indicates regression coefficient. SE indicates standard error. * indicates ***p*** < 0.05, ** indicates ***p*** < 0.01, *** indicates ***p*** < 0.001. Each specification was tested separately with adjustment for confounders in the first model (Models 1, 3, 5, 7) and mediators in the second (Models 2, 4, 6, 8)

### Mediation analyses

**Fig. 1:**
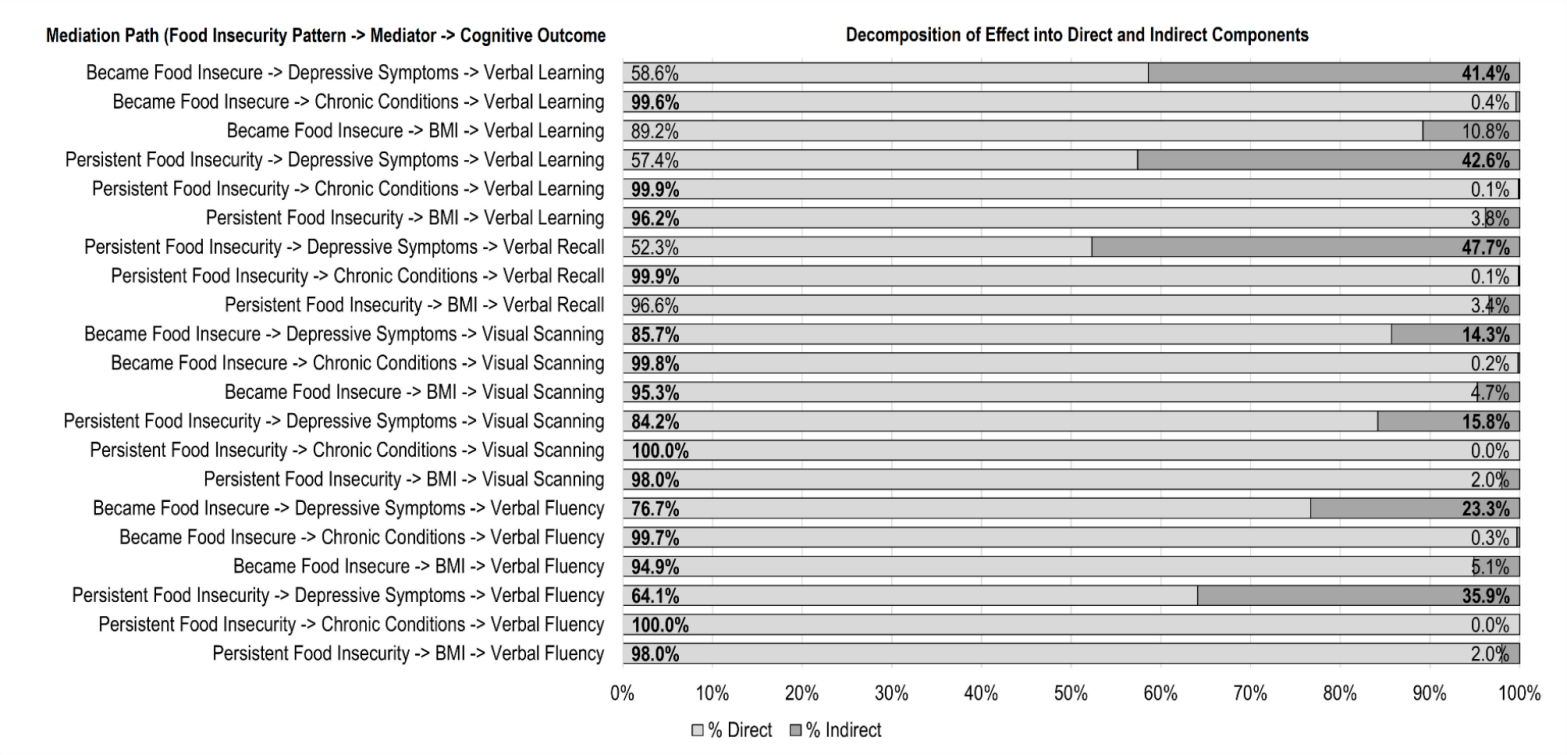
Decomposition of food insecurity-cognition associations into direct and indirect components Note: Bold numbers indicate statistical significance of direct/indirect effects. Mediation is tested using Karlson, Holm, and Breen methods. Each row represents a mediation path, which are listed on the left-hand side of the figure as “food insecurity pattern -> mediator -> cognitive outcome.” Each mediation path is tested separately to allow for testing of statistical significance of each mediator. Indirect percentages across rows may not necessarily be summed due to correlation of mediating variables. Mediation is only tested for food insecurity effects that were significant in regression analyses (Table 2). Thus, mediation results for associations between becoming food insecure and Verbal Recall are not tested. Effects are based on linear regressions predicting cognitive scores controlling for confounding variables (age, gender, years of education, locality size, marital status, social capital, household residents, speaking an indigenous dialect, income and wealth deciles). Mediation tests involving BMI include the missing BMI flag as an additional control variable

We then formally tested the statistical significance of mediation (indirect) effects and what mediators were driving indirect effects, which we illustrate in Fig. 1. For each instance in our regression analyses in Table 2 in which we observed a significant association between a food insecurity pattern and cognition, we proceeded to test what percentage of the association between the food insecurity pattern and the cognitive outcome is “direct”, which represents the percent of the total association between the food insecurity pattern and cognition that is unexplained by the mediating variable, and what percent is “indirect”, which represents the percent of the total association between the food insecurity pattern and cognition that is explained by the mediating variable. For each significant association that we observe in regression results, we test the mediation effect through each proposed mediator (depressive symptoms, chronic conditions, and BMI) separately as this allows us to test whether each proposed mediator significantly mediates the association between food insecurity and cognition. Bolded percentages are used to denote whether direct and indirect effects are statistically significant. Our primary interest is in the significance of the indirect effect as this implies statistically significant mediation.

For Verbal Learning, we found that effects of food insecurity were significantly mediated by depressive symptoms. Around 40% of the associations between becoming food insecure and persistent food insecurity with Verbal Learning were explained by an indirect effect through depressive symptoms. By contrast, no significant indirect effects through BMI nor chronic conditions were observed. Similarly, depressive symptoms significantly mediated the association between persistent food insecurity and Verbal Recall, with nearly half of the association being attributed to an indirect effect through depressive symptoms, whereas BMI and chronic conditions did not significantly mediate the association.

Mediation results for Visual Scanning and Verbal Fluency largely mirrored those for Verbal Learning. For Visual Scanning, 14.3% of the effect of becoming food insecure and 15.8% of the effect of persistent food insecurity were explained by depressive symptoms. Food insecurity also seemed to relate with Verbal Fluency indirectly through depressive symptoms with 23.3% and 35.9% of the association between becoming food insecure and persistent food insecurity with Verbal Fluency, respectively, being attributed to an indirect effect through depressive symptoms. There was no evidence that food insecurity impacted Visual Scanning or Verbal Fluency indirectly through chronic conditions or BMI.

## Discussion

As population aging has progressed in Mexico, the need to understand risk factors for poor health has increased in step. Growing research emphasizes the need to address old age poverty, and its related health effects, given that older Mexican adults account for over half of families in poverty [3]. The current study focused on late-life food insecurity, to which individuals living in poverty are more likely to be exposed and examined whether patterns of food insecurity were associated with cognitive function. Our findings suggest that late-life food insecurity is related with worse cognitive outcomes, but with effects that are conditional on the cognitive tasks assessed and the timing of food insecurity in old age. Our findings are consistent with prior work using the international network of Health and Retirement Study (HRS) sister studies that have reported associations between food insecurity and worse cognitive outcomes in India, Europe, Mexico, and the United States [23, 27, 29, 32, 39].

Prior research in Mexico has investigated potential links between food insecurity and cognition among older adults and has found late-life food insecurity to relate with poorer Visual Scanning performance [29] and food availability to relate with better immediate and delayed recall [7]. The current study extends the scientific understanding of food insecurity and cognition in Mexico in important ways. First, our analyses pushed scientific research on food insecurity and cognition forward by formally testing the potential mechanisms through which effects of food insecurity may operate. We observed that associations between becoming food insecure and persistent food insecurity and cognition are mediated by depressive symptoms. Second, past studies of late-life food insecurity and cognition have largely only considered measurements of food insecurity at one point in late-life and no studies in Mexico have considered changes in food insecurity status in late-life. This study extends past work by acknowledging the cyclical nature of food insecurity by examining how patterns of late-life food insecurity over time relate with cognition, allowing a deeper understanding of the roles of timing and duration of late-life food insecurity.

Persistent food insecurity was associated with worse performance across all cognitive outcomes whereas becoming food insecure was associated with all cognitive outcomes except Verbal Recall. Although persistent food insecurity, but not becoming food insecure, was related with Verbal Recall, our sensitivity analyses showed no difference in any cognitive task between becoming food insecure and being persistently food insecure. These results indicate that persistence of food insecurity is not significantly more detrimental to cognition than currently experiencing food insecurity. Lu and colleagues [43] observed similar results when comparing duration of food insecurity and cognition. Although they did not compare the persistently food insecure with those who became food insecure, the cognitive scores of those with persistent food insecurity only differed significantly from those who were persistently food secure for one cognitive task. On the other hand, becoming food insecure was associated with lower cognitive scores in almost all cognitive tasks assessed [43]. The shorter time-points (five or fewer years) between the two assessments of food insecurity in both our study and Lu and colleagues [43] may help explain these results. There may be a greater impact of persistent food insecurity compared to becoming food insecure if food insecurity persisted over a longer period. These findings may also suggest that current exposure to food insecurity during the time of the survey could affect one’s ability to respond to the cognitive tasks. Our results also showed that former experiences of food insecurity were not associated with worse cognitive outcomes compared to persistent food security. Similarly, Lu and colleagues [43] also observed no differences in cognitive tasks between those who become food secure compared to the persistently food secure. This result also indicates that it is the timing of the effect that matters more than the duration of food insecurity in old age.

We note that the parameter estimates for both persistent food insecurity and becoming food insecure were smallest in Verbal Recall models and that becoming food insecure did not relate with Verbal Recall. Past studies suggest that effects of food insecurity may differ across cognitive domains, with executive function being most negatively affected [25, 28, 31]. Visual Scanning tasks involve multiple cognitive domains including attention and executive function [69] and Verbal Fluency tasks involve domains including executive function and language [70]. Furthermore, deficits in Verbal Learning performance correlate with executive function deficits [71], which may be explained by impairments in executive function affecting information storage/retrieval [72]. Thus, effects on these tasks may be related to executive dysfunction. The notable effects of food insecurity on executive function may be explained by the prefrontal cortex, a region instrumental to executive function, being especially vulnerable to stressors such as food insecurity [73, 74]. Recent work using functional magnetic resonance imaging (fMRI) has also noted abnormal correlations between activity in the frontoparietal network (FPN) and default mode network (DMN) in individuals experiencing food insecurity, which may explain impairment in executive function [26].

Our analyses extend past work by considering multiple potential mediators (depressive symptoms, BMI, and chronic conditions). Indirect effects were primarily attributable to depressive symptoms with between 14% and 48% of the total associations between food insecurity and cognition being mediated through depressive symptoms. Past work has established depression as a mediator connecting food insecurity to cognition [33, 43]. Food insecurity may induce both stress and depression in behavioral and biological ways. Unpredictability of food activates stress responses that promote depression [75]. Procuring food in socially improper ways may also generate feelings of alienation, powerlessness, shame, and guilt, all of which are associated with depressive symptoms [41, 76–78]. Food insecurity is also related with lower diet quality including increased consumption of fat and refined sugars and lessened consumption of fruits and vegetables [79], which are associated with psychological problems such as depression [80].

There have been a few policy efforts to reduce food insecurity in Mexico. Anti-poverty programs in Mexico, such as the conditional cash transfer program *PROSPERA* and the non-contributory pension program *70 y Más* have been designed with improving nutrition and alleviating hunger in mind. Evaluations have shown positive impacts including reductions in food vulnerability associated with *70 y Más* [81] and diminished household food insecurity associated with *PROSPERA* [82]. Policies such as these may help decrease the burden of food insecurity and its negative impact on cognition.

Our analyses have limitations. First, reverse causality may be present as lower cognitive ability in early adulthood and midlife may impede one’s ability to obtain stable high paying work, causing greater likelihood of food insecurity. Second, our food insecurity measure may not capture complexities of food insecurity, including severity. Food insecurity questions only refer to experiences at any time in the two years prior to interviews. This leaves unobserved variation in when, and how often, in the past two years food insecurity occurred and whether it occurred before the study such as in midlife. Future work should assess severity and temporal patterning of food insecurity on a fine-grained timeframe (e.g., monthly, weekly), include assessments of food insecurity and cognition from old age along with early adulthood and midlife, and consider more complex patterns of experiencing food insecurity over time. Furthermore, our measure of food insecurity was based on two questions. We note that this approach is consistent with prior work [41], but given the reliance on only two questions we caution readers that this approach may result in more misclassification of food insecurity compared to more extensive batteries such as the Latin American and Caribbean Food Security Scale (ELCSA) [83].

Further research should consider the temporal ordering of variables in mediation models. Given that we evaluate food insecurity in 2015 and 2018 waves, and each involves a two-year lookback period, we can be more confident that measures of food insecurity precede measurement of cognition, which is assessed during the 2018 survey. However, we include mediators from the 2018 wave too, making the ordering of mediating variables and cognition less certain. Regarding depressive symptoms, for which we found the clearest suggestion of mediation, there is substantial research suggesting depression to increase risk of poor cognition, yet the relationship may be bidirectional with limitations in certain facets of cognitive ability related with a worsening course of depressive symptoms [84]. An alternative approach has been employed by Lu and colleagues (2023) involving assessment of change in food insecurity in Time 1 and 2, mediators in Time 3, and cognition in Time 4. Although this approach may be feasible with MHAS data, we opted for our approach as using only measures of food insecurity from several waves prior to measurement of cognition precludes our ability to evaluate potential effects of *recent* food insecurity as measures of food insecurity would all be two or more waves before measures of cognition. Nevertheless, our design in which mediators and cognition are assessed simultaneously prevents causal conclusions regarding mediation. Last, although we assess food insecurity at multiple timepoints, we only analyze cognition at one point. Future work should consider how timing and duration of food insecurity relates with cognitive change. Despite these limitations, the MHAS data provides several strengths including the use a large nationally representative sample, a comprehensive array of confounding and mediating variables, cognitive assessments spanning multiple cognitive domains, and assessment of food insecurity through old age.

These results have public health and policy implications. Eliminating food insecurity remains a vital public health goal, particularly in low- and middle-income countries where food insecurity is more common. Our results suggest that food insecurity is related with worse cognitive function, particularly with executive function. This suggests that, in addition to alleviating suffering, public health interventions and policy that address late-life food insecurity may have benefits that extend to better health and cognitive function. Importantly, food insecurity is modifiable and identification of modifiable risk factors for poor cognitive outcomes in old age is critical in Mexico [4] and other low- and middle-income countries where populations continue to age and the number of people living with dementia is expected to expand in coming decades [85]. Interventions and policies should promote reliable access to healthy foods for individuals living in poverty.

## Data Availability

The data files and documentation of data used in this study are public use and available at the Mexican Health and Aging Study website at www.MHASweb.org. As per the data use agreement of the Mexican Health and Aging Study, the authors may “not transfer MHAS public release data to a third party(-ies), with the exception of staff or students for whom said user is directly responsible.”

## References

[1] Aguila E, Diaz C, Fu M, Kapteyn A, Pierson A. Living Longer in Mexico: Income Security and Health. 2012. 10.7249/rhq-01-04-01 PMC4945250 28083208

[2] Wong R, Palloni A. Aging in Mexico and Latin America. In: Uhlenberg P, editor. International Handbook of Population Aging. Springer Netherlands; 2009. pp. 231–52.

[3] Angel JL, Vega W, López-Ortega M (2017). Aging in Mexico: Population trends and Emerging issues. Gerontologist.

[4] Alzheimer’s Disease International, Bupa. Dementia in the Americas: Current and Future Cost and Prevalence of Alzheimer’s Disease and Other Dementias. 2013.

[5] Díaz-Venegas C, Samper-Ternent R, Michaels-Obregón A, Wong R (2019). The effect of educational attainment on cognition of older adults: results from the Mexican Health and Aging Study 2001 and 2012. Aging Ment Health.

[6] Torres JM, Rizzo S, Wong R (2018). Lifetime socioeconomic status and late-life Health trajectories: longitudinal results from the Mexican Health and Aging Study. J Gerontol B Psychol Sci Soc Sci.

[7] Aguila E, Casanova M (2020). Short-term impact of income on cognitive function: evidence from a sample of Mexican older adults. J Aging Health.

[8] Al Hazzouri AZ, Haan MN, Galea S, Aiello AE (2011). Life-course exposure to early socioeconomic environment, education in relation to late life cognitive function among older mexicans and Mexican americans. J Aging Health.

[9] Food and Agriculture Organization. Draft Declaration of the World Summit on Food Insecurity. 2009.

[10] Food and Agriculture Organization. The State of Food Insecurity and Nutrition around the World 2021. 2021.

[11] Smith MD, Kassa W, Winters P (2017). Assessing food insecurity in Latin America and the Caribbean using FAO’s Food Insecurity Experience Scale. Food Policy.

[12] Zilak J, Gundersen C. The health consequences of senior hunger in the United States: Evidence from the 1999–2014 NHANES. Prepared for Feeding America and the National Foundation to End Senior Hunger. 2017.

[13] Pourmotabbed A, Moradi S, Babaei A, Ghavami A, Mohammadi H, Jalili C (2020). Food insecurity and mental health: a systematic review and meta-analysis. Public Health Nutr.

[14] Smith L, Il Shin J, McDermott D, Jacob L, Barnett Y, López-Sánchez GF (2021). Association between food insecurity and depression among older adults from low- and middle-income countries. Depress Anxiety.

[15] Pak T-Y, Kim G (2021). Association of Food Insecurity with allostatic load among older adults in the US. JAMA Netw Open.

[16] Bishop NJ, Wang K (2018). Food insecurity, comorbidity, and mobility limitations among older U.S. adults: findings from the Health and Retirement Study and Health Care and Nutrition Study. Prev Med.

[17] Petersen CL, Brooks JM, Titus AJ, Vasquez E, Batsis JA (2019). Relationship between Food Insecurity and Functional limitations in older adults from 2005–2014 NHANES. J Nutr Gerontol Geriatr.

[18] Cai J, Bidulescu A (2023). The association between chronic conditions, COVID-19 Infection, and food insecurity among the older US adults: findings from the 2020–2021 National Health interview survey. BMC Public Health.

[19] Hadfield-Spoor M, Avendano M, Loopstra R (2022). Food insecurity among disabled adults. Eur J Public Health.

[20] Heflin CM, Altman CE, Rodriguez LL (2019). Food insecurity and disability in the United States. Disabil Health J.

[21] McMichael AJ, McGuinness B, Lee J, Minh HV, Woodside JV, McEvoy CT (2022). Food insecurity and brain health in adults: a systematic review. Crit Rev Food Sci Nutr.

[22] Na M, Dou N, Ji N, Xie D, Huang J, Tucker KL (2020). Food insecurity and cognitive function in Middle to older Adulthood: a systematic review. Adv Nutr.

[23] Cohn-Schwartz E, Weinstein G (2020). Early-life food deprivation and cognitive performance among older europeans. Maturitas.

[24] Frith E, Loprinzi PD (2018). Food insecurity and cognitive function in older adults: brief report. Clin Nutr.

[25] Gao X, Scott T, Falcon LM, Wilde PE, Tucker KL (2009). Food insecurity and cognitive function in Puerto Rican adults. Am J Clin Nutr.

[26] Guerithault N, McClure SM, Ojinnaka CO, Braden BB, Bruening M (2022). Resting-State Functional Connectivity Differences in College Students with and without Food Insecurity. Nutrients.

[27] Kumar S, Bansal A, Shri N, Nath NJ, Dosaya D (2021). Effect of food insecurity on the cognitive problems among elderly in India. BMC Geriatr.

[28] Portela-Parra ET, Leung CW (2019). Food Insecurity is Associated with Lower Cognitive Functioning in a National Sample of older adults. J Nutr.

[29] Saenz JL, Kessler J, Nelson E (2022). Food Insecurity across the life-course and cognitive function among older Mexican adults. Nutrients.

[30] Na M, Dou N, Brown MJ, Chen-Edinboro LP, Anderson LR, Wennberg A (2023). Food Insufficiency, Supplemental Nutrition Assistance Program (SNAP) Status, and 9-Year trajectory of cognitive function in older adults: the Longitudinal National Health and Aging trends Study, 2012–2020. J Nutr.

[31] Wong JC, Scott T, Wilde P, Li Y-G, Tucker KL, Gao X (2016). Food Insecurity is Associated with subsequent cognitive decline in the Boston Puerto Rican Health Study. J Nutr.

[32] Lu P, Kezios K, Jawadekar N, Swift S, Vable A, Zeki Al Hazzouri A (2023). Associations of Food Insecurity and memory function among Middle to older–aged adults in the Health and Retirement Study. JAMA Netw Open.

[33] Cai J, Bidulescu A (2023). The association between food insecurity and cognitive impairment among the US adults: the mediation role of anxiety or depression. J Affect Disord.

[34] Koyanagi A, Veronese N, Stubbs B, Vancampfort D, Stickley A, Oh H (2019). Food Insecurity is Associated with mild cognitive impairment among middle-aged and older adults in South Africa: findings from a nationally Representative Survey. Nutrients.

[35] Onadja Y, Atchessi N, Soura BA, Rossier C, Zunzunegui M-V (2013). Gender differences in cognitive impairment and mobility disability in old age: a cross-sectional study in Ouagadougou, Burkina Faso. Arch Gerontol Geriatr.

[36] Srivastava S, Muhammad T (2022). Rural-urban differences in food insecurity and associated cognitive impairment among older adults: findings from a nationally representative survey. BMC Geriatr.

[37] Zhang Z, Gu D, Hayward MD (2008). Early life influences on cognitive impairment among Oldest Old Chinese. J Gerontol Ser B.

[38] Momtaz YA, Haron SA, Hamid TA, Ibrahim R, Masud J (2014). Does food insufficiency in childhood contribute to Dementia in later life?. Clin Interv Aging.

[39] Pengpid S, Peltzer K (2023). Food insecurity and health outcomes among community-dwelling middle-aged and older adults in India. Sci Rep.

[40] Arenas DJ, Thomas A, Wang J, DeLisser HM (2019). A systematic review and Meta-analysis of Depression, anxiety, and Sleep disorders in US adults with Food Insecurity. J Gen Intern Med.

[41] Bergmans RS, Wegryn-Jones R (2020). Examining associations of food insecurity with major depression among older adults in the wake of the great recession. Soc Sci Med.

[42] Diniz BS, Butters MA, Albert SM, Dew MA, Reynolds CF (2013). Late-life depression and risk of vascular Dementia and Alzheimer’s Disease: systematic review and meta-analysis of community-based cohort studies. Br J Psychiatry J Ment Sci.

[43] Lu P, Kezios K, Yaffe K, Kim S, Zhang A, Milazzo FH (2023). Depressive symptoms mediate the relationship between sustained food insecurity and cognition: a causal mediation analysis. Ann Epidemiol.

[44] Pérez-Escamilla R, Villalpando S, Shamah-Levy T, Méndez-Gómez Humarán I (2014). Household food insecurity, Diabetes and Hypertension among Mexican adults: results from Ensanut 2012. Salud Pública México.

[45] Seligman HK, Bindman AB, Vittinghoff E, Kanaya AM, Kushel MB (2007). Food Insecurity Is Associated with Diabetes Mellitus: results from the National Health Examination and Nutrition Examination Survey (NHANES) 1999–2002. J Gen Intern Med.

[46] Seligman HK, Laraia BA, Kushel MB (2010). Food Insecurity is Associated with Chronic Disease among low-income NHANES participants. J Nutr.

[47] Canavan M, O’Donnell MJ. Hypertension and cognitive impairment: a review of mechanisms and Key concepts. Front Neurol. 2022;13. 10.3389/fneur.2022.821135 PMC8855211 35185772

[48] McGrath ER, Beiser AS, O’Donnell A, Himali JJ, Pase MP, Satizabal CL (2022). Determining vascular risk factors for Dementia and Dementia Risk Prediction Across Mid- to later life: the Framingham Heart Study. Neurology.

[49] Brown AGM, Esposito LE, Fisher RA, Nicastro HL, Tabor DC, Walker JR (2019). Food insecurity and obesity: research gaps, opportunities, and challenges. Transl Behav Med.

[50] Dhurandhar EJ (2016). The food-insecurity obesity Paradox: A Resource Scarcity Hypothesis. Physiol Behav.

[51] Kandapan B, Pradhan I, Pradhan J (2022). Food Insecurity and Malnutrition among Indian older adults: findings from Longitudinal Ageing Study in India, 2017-18. J Popul Ageing.

[52] Moradi S, Mirzababaei A, Dadfarma A, Rezaei S, Mohammadi H, Jannat B (2019). Food insecurity and adult weight abnormality risk: a systematic review and meta-analysis. Eur J Nutr.

[53] Sabia S, Kivimaki M, Shipley MJ, Marmot MG, Singh-Manoux A (2009). Body mass index over the adult life course and cognition in late midlife: the Whitehall II Cohort Study. Am J Clin Nutr.

[54] Ribar DC, Hamrick KS, editors. Dynamics of Poverty and Food Sufficiency. 2003.

[55] Richard E, Reitz C, Honig LH, Schupf N, Tang MX, Manly JJ (2013). Late-Life Depression, mild cognitive impairment, and Dementia. JAMA Neurol.

[56] Rock PL, Roiser JP, Riedel WJ, Blackwell AD (2014). Cognitive impairment in depression: a systematic review and meta-analysis. Psychol Med.

[57] Saenz JL, Garcia MA, Downer B (2020). Late life depressive symptoms and cognitive function among older Mexican adults: the past and the present. Aging Ment Health.

[58] Chen R, Williams DR, Nishimi K, Slopen N, Kubzansky LD, Weuve J (2022). A life course approach to understanding stress exposures and cognitive function among middle-aged and older adults. Soc Sci Med.

[59] Smith MD, Rabbitt MP, Coleman- Jensen A (2017). Who are the World’s Food Insecure? New evidence from the Food and Agriculture Organization’s Food Insecurity Experience Scale. World Dev.

[60] Gaitán-Rossi P, Vilar-Compte M, Teruel G, Pérez-Escamilla R (2021). Food insecurity measurement and prevalence estimates during the COVID-19 pandemic in a repeated cross-sectional survey in Mexico. Public Health Nutr.

[61] MHAS Mexican Health and Aging Study. Data Files and Documentation (public use): Mexican Health and Aging Study, 2012, 2015, and 2018. 2012.

[62] Wong R, Michaels-Obregon A, Palloni A (2017). Cohort Profile: the Mexican Health and Aging Study (MHAS). Int J Epidemiol.

[63] Mejía-Arango S, Wong R, Michaels-Obregón A (2015). Normative and standardized data for cognitive measures in the Mexican Health and Aging Study. Salud Pública México.

[64] Mejia-Arango S, Gutierrez LM (2011). Prevalence and incidence rates of Dementia and cognitive impairment no Dementia in the Mexican Population Data from the Mexican Health and Aging Study. J Aging Health.

[65] Magaña-Lemus D, Ishdorj A, Rosson CP, Lara-Álvarez J (2016). Determinants of household food insecurity in Mexico. Agric Food Econ.

[66] Radloff LS (1977). The CES-D scale A self-report depression scale for research in the general population. Appl Psychol Meas.

[67] Avila-Funes JA, Gutierrez-Robledo LM (2004). Validity of height and weight self-report in Mexican adults: results from the national health and aging study. J Nutr Health Aging.

[68] Karlson KB, Holm A (2011). Decomposing primary and secondary effects: a new decomposition method. Res Soc Stratif Mobil.

[69] Peña-González P, Mondragón-Maya A, Silva-Pereyra J, Roa-Rojas P (2020). Cognitive Reserve and executive functions in adults with type 2 Diabetes. J Diabetes Res.

[70] Gustavson DE, Panizzon MS, Franz CE, Reynolds CA, Corley RP, Hewitt JK (2019). Integrating Verbal fluency with executive functions: evidence from Twin studies in Adolescence and Middle Age. J Exp Psychol Gen.

[71] Duff K, Schoenberg MR, Scott JG, Adams RL (2005). The relationship between executive functioning and verbal and visual learning and memory. Arch Clin Neuropsychol.

[72] Della Sala S, Laiacona M, Spinnler H, Trivelli C (1993). Autobiographical recollection and frontal damage. Neuropsychologia.

[73] Girotti M, Adler SM, Bulin SE, Fucich EA, Paredes D, Morilak DA (2018). Prefrontal cortex executive processes affected by stress in health and Disease. Prog Neuropsychopharmacol Biol Psychiatry.

[74] Shansky R, Lipps J. Stress-induced cognitive dysfunction: hormone-neurotransmitter interactions in the prefrontal cortex. Front Hum Neurosci. 2013;7. 10.3389/fnhum.2013.00123 PMC3617365 23576971

[75] Kessler RC (1997). The effects of Stressful Life events on Depression. Annu Rev Psychol.

[76] Bernal J, Frongillo EA, Jaffe K (2016). Food insecurity of children and shame of others knowing they are without Food. J Hunger Environ Nutr.

[77] Gilbert P. Depression: the evolution of powerlessness. Routledge; 2016.

[78] Nanama S, Frongillo EA (2012). Altered social cohesion and adverse psychological experiences with chronic food insecurity in the non-market economy and complex households of Burkina Faso. Soc Sci Med.

[79] Leung CW, Epel ES, Ritchie LD, Crawford PB, Laraia BA (2014). Food insecurity is inversely associated with diet quality of lower-income adults. J Acad Nutr Diet.

[80] Lang UE, Beglinger C, Schweinfurth N, Walter M, Borgwardt S (2015). Nutritional aspects of Depression. Cell Physiol Biochem.

[81] Juarez L, Pfutze T (2020). Can non-contributory pensions decrease food vulnerability? The case of Mexico. Empir Econ.

[82] Saldivar-Frausto M, Unar-Munguía M, Méndez-Gómez-Humarán I, Rodríguez-Ramírez S, Shamah-Levy T (2022). Effect of a conditional cash transference program on food insecurity in Mexican households: 2012–2016. Public Health Nutr.

[83] FAO. Escala Latinoamericana y Caribeña de Seguridad Alimentaria (ELCSA). 2012.

[84] van den Kommer TN, Comijs HC, Aartsen MJ, Huisman M, Deeg DJH, Beekman ATF (2013). Depression and Cognition: how do they interrelate in Old Age?. Am J Geriatr Psychiatry.

[85] Alzheimer’s Disease International. World Alzheimer Report 2015. The Global Impact of Dementia. An analysis of prevalence, incidence, cost and trends. 2015.

